# Efficacy in Predicting Negative Appendectomy Rates in Operated Acute Appendicitis Patients Using the Raja Isteri Pengiran Anak Saleha Appendicitis (RIPASA) Score Versus Modified Alvarado Score

**DOI:** 10.7759/cureus.37873

**Published:** 2023-04-20

**Authors:** Suhas Devanathan, Swati G Deshpande, Darshana Tote, Sandip Shinde

**Affiliations:** 1 General Surgery, Jawaharlal Nehru Medical College, Datta Meghe Institute of Higher Education and Research, Wardha, IND; 2 General Surgery, Chhatrapati Multispeciality Hospital, Hingoli, IND

**Keywords:** clinical scoring method, ripasa scoring system, modified alvarado scoring system, negative appendicectomies, acute appendicitis

## Abstract

Introduction

Acute appendicitis is the commonest abdominal surgical emergency globally. The most accepted management of acute appendicitis is surgical, either open or laparoscopic appendectomy. Overlapping clinical presentations with many genitourinary and gynecological conditions lead to difficulty in accurate diagnosis, making negative appendectomies an unwanted reality. With the advancement in technology, there have been constant efforts to minimize negative appendectomy rates (NAR) using imaging modalities like USG of the abdomen and the gold-standard imaging test, the contrast-enhanced computed tomography of the abdomen. Due to the cost incurred and the lesser availability of such imaging modalities and needed expertise in resource-poor settings, various clinical scoring systems were devised to accurately diagnose acute appendicitis and thereby decrease NAR. We conducted our study to determine the NAR between the Raja Isteri Pengiran Anak Saleha Appendicitis score (RIPASA) and the modified Alvarado (MA) scoring methods.

Methods

A prospective observational analytical study was conducted, including 50 patients presenting to our hospital with acute appendicitis and who underwent emergency open appendectomy. The need to operate was decided by the treating surgeon. Patients were stratified by both scores; the pre-operative scores were noted and were later compared with the histopathological diagnosis.

Results

A total of 50 clinically diagnosed patients with acute appendicitis were evaluated utilizing the RIPASA and the MA scores. The NAR was 2% using the RIPASA score vs 10% with the MA score. The sensitivity was 94.11% vs 70.58% (p<0.0001), the specificity was 93.75% vs 68.75% (p<0.0001), the positive predictive value (PPV) of 96.96% vs 82.75% (p<0.001), the negative predictive value (NPV) of 88.23% vs 52.38% (p<0.001), and NAR of 2% vs 10% (p<0.0001) in the RIPASA vs MA scoring method, respectively.

Conclusions

RIPASA score is highly efficacious and statistically significant in diagnosing acute appendicitis with higher PPV at higher scores and higher NPV with lower scores leading to decreased NAR compared with MA score.

## Introduction

The incidence of acute appendicitis is high among the surgical emergencies presenting to the hospital. The common clinical features of many other gastrointestinal, obstetric-gynecological, and genitourinary conditions make the exact diagnosis of acute appendicitis often difficult [1]. Without prompt surgical intervention, there is an increased predisposition to sequelae such as appendicular perforation with peritonitis, intraabdominal abscess formation, and septicemia leading to an extended hospital stay and monetary implications. This may be associated with significant mortality (1-5%) and morbidity (10%) [2]. The negative appendectomy rate (NAR), a well-known consequence of appendectomy, in histopathological negative appendicitis specimens, varies between 6% to 44% in the literature based solely on clinical diagnosis and between 0.7% and 17% using imaging modalities like USG and contrast-enhanced computed tomography (CECT) of the abdomen and pelvis. The acceptable NAR was 20% [2] before the advent of the CECT abdomen but now is expected to be below 2% [3].

The Dutch College of Surgeons in their 2010 guidelines stated that USG or CECT scan is recommended to confirm the diagnosis before surgery in patients suspected of acute appendicitis [4]. However, the imaging modalities may not always be available, accessible, or affordable especially in resource-poor settings due to poor patients, lack of trained radiologists, lack of electricity, etc., necessitating the presence of easily applicable clinical scoring systems to diagnose acute appendicitis and refute other conditions with increased confidence levels. Thus, this has led to the emergence of various clinical scores over the years to diagnose acute appendicitis [5]. There has been a constant strive toward reducing the number of negative appendicectomies and, hence, the NAR. Kalan et al. in 1994 described the modified Alvarado (MA) score [6] with well-proven efficacy in the Western population but not all of the Asian and middle eastern populations. The Raja Isteri Pengiran Anak Saleha Appendicitis (RIPASA) score was developed by Chong et al. (2010) for diagnosing appendicitis and reducing NAR, using data on 312 patients, and found it more predictive than the MA score in these populations [7]. This study was done to compare NAR between the MA score and the RIPASA system.

## Materials and methods

A prospective observational analytical study was done at Acharya Vinoba Bhave Rural Hospital and Jawaharlal Nehru Medical College, Sawangi, Wardha, involving 50 patients between 2018 and 2020. Approval from the Ethics Committee (DMIMS(DU)/IEC/2018- 19/7413) was taken. Patients who presented with acute appendicitis were evaluated and operated upon at the discretion of the surgeon who was a professor in the Department of General Surgery with more than 10 years of experience. All consenting patients greater than 13 years were included. Patients who were less than the age of 13 years, had a known history of confounding gynecological or genitourinary conditions, or did not consent to the study were excluded. All the included patients were stratified into definite, high probability, low probability, and unlikely scores by the MA score [6] (Table 1) and the RIPASA score [7] (Table 2).

**Table 1:** MA score RIF: right iliac fossa Score: > 8: definite appendicitis 6-7: high probability of appendicitis 5-6: low probability of appendicitis < 5: unlikely of appendicitis [6]

Parameter	Score
Symptoms	
Migratory RIF pain	1
Nausea/vomiting	1
Anorexia	1
Signs	
Tenderness in RIF	2
Rebound tenderness in RIF	1
Elevated temperature > 37.5 ▫ C	1
Laboratory findings	
Leucocytosis	2
Total	9

**Table 2:** RIPASA score RIF: right iliac fossa, WBC: white blood cell, NRIC: national registration identity card Score: 12: definite appendicitis 7.5-11.5: high probability of appendicitis 5-7.5: low probability of appendicitis < 5: unlikely of appendicitis [7]

Patient’s demography	Score
Female	0.5
Male	1
Age < 39.9 years	1
Age > 40 years	0.5
Symptoms	
RIF pain	0.5
Pain migration to RIF	0.5
Anorexia	1
Nausea and vomiting	1
Duration of symptoms < 48 hours	1
Duration of symptoms > 48 hours	0.5
Signs	
RIF tenderness	1
Guarding	2
Rebound tenderness	1
Rovsing’s sign	2
Fever > 37^0^C	1
Investigations	
Raised WBC count	1
Negative urinalysis	1
Additional scores	
Foreign NRIC (non-Iraqi patients)/non-Asian	1

Sample size calculation was done according to the following formula used to compare the two methods with known accuracy:

N = [(Z_(1-alpha/2)_+Z_beta_)^2^ (P_1_ (1-P_1_) + (P_2_(1-P_2)_] / (P_2_- P_1_)^2^

Z_alpha/2_ = 2.807

alpha = type I error at 0.05%

Z_beta_ = 1.28

beta = type II error at 10%

Primary variable: prediction of NAR

P1 = 48 % accuracy for MA and P2 = 88% for RIPASA (as per ref article [8]).Clinically relevant difference (P2-P1) = 40 %

Minimum sample size N = (2.807 + 1.28 )2 (0.48) (1-0.48) + (0.88) (1-0.88)/ (0.4 )2 = 37.

The total sample size required = 37.

We included 50 patients in our study. Those with definite and high probability were considered positive, and the ones with low probability and unlikely scores were considered negative as per the scoring methods. The clinician who admitted and made the decision regarding surgical intervention in both low and high probability scores were professors in the Department of General Surgery with a minimum experience of 10 years. The resected specimen of appendices was sent for histopathology and noted either positive or negative for acute appendicitis. Both scoring systems were individually studied to estimate the false positive cases compared with histopathology results. Sensitivity, specificity, negative predictive value (NPV), positive predictive value (PPV), and NAR were calculated for both scoring systems. Both scores were compared with each other to determine the efficacy of the RIPASA versus the MA scoring method using the chi-square test and utilizing the SPSS Software Version 27.0.1.0 (IBM Corp. Released 2020. IBM SPSS Statistics for Windows, Version 27.0. Armonk, NY: IBM Corp).

## Results

A total of 50 operated patients with acute appendicitis were included, and the scores were recorded according to the clinical scoring systems. A total of 68% (34/50) of patients were noted to have an accurate diagnosis, while 32% (16/50) of patients did not have acute appendicitis.

The most common age group affected was the 15 to 30 years age group with 46% (23/50) patients, followed by 26% (13/50) patients in the 31 to 45 years, 14%(7/50) in the 46 to 60 years, and 8% (4/50) in the less than 15 years. The least common age group affected was 61 to 75 years with 6% (3/50) patients. Thus, the incidence is more common in young adults compared to extremes of age. Males were twice (n=33, 66%) as commonly affected compared with females (n=17, 34%).

In this study, as shown in Table 3, the MA score was more than 9, i.e., definite in 30% (15/50) patients, and 28%(14/50) patients had a score in the range between 7 and 8 (high probability). There were 42% (21/50) of patients who had a score between 5 and 6. The patients with unlikely scores were not found to be surgical candidates by the treating surgeon clinically.

**Table 3:** Results of the MA score MA: modified Alvarado

MA score	Histopathology positive	Histopathology negative	Total
Definite score (9)	15	0	15
High probability score (7-8)	9	5	14
Low probability score (5-6)	10	11	21
Unlikely score (<5)	0	0	0
Total	34	16	50

As shown in Table 4, it was found that 48% (24/50) of patients had high scores, i.e., were true positive patients, whereas 10% (5/50) of patients were false positive, i.e., negative appendectomies. A total of 22% (11/50) of patients had true negative scores, i.e., negative appendectomies with low probability scores between 5 and 6 (low probability).

**Table 4:** Correlation of high and low MA scores with histopathology MA: modified Alvarado, TP: true positive, FP: false positive, FN: false negative, TN: true negative

MA score	Histopathology positive	Histopathology negative	Total
High score	24 (TP)	5 (FP)	29
Low score	10 (FN)	11 (TN)	21
Total	34	16	50

As shown in Table 5, it was noted that 56% (28/50) of patients had a definite score of more than 12 according to the RIPASA score, and all were true positive patients as per histopathology. There were 10% (5/50) of patients who had a score in the range between 7.5 and 11.5 (high probability) of which 8% (4/50) of patients were true positive and 2% (1/50) were false positive. There were 34% (17/50) of patients who had a score between 5 and 7.5 (low probability), of which 4% (2/50) of patients were true positive and 30% (15/50) were true negative. The patients with unlikely scores were not found to be surgical candidates by the treating surgeon clinically.

**Table 5:** Results of the RIPASA score RIPASA: Raja Isteri Pengiran Anak Saleha Appendicitis

RIPASA score	Histopathology positive	Histopathology negative	Total
Definite score (12)	28	0	15
High probability score (7.5-11.5)	4	1	14
Low probability score (5-7.5)	2	15	21
Unlikely score (<5)	0	0	0
Total	34	16	50

As shown in Table 6, it was found that 64% (32/50) of patients had high scores, i.e., true positive cases, whereas only 2% (1/50) had a negative appendectomy, i.e., false positive score, versus 30% (15/50) who had negative appendectomy with low probability scores. This shows a high PPV for high scores and a high NPV for low probability scores utilizing the RIPASA score.

**Table 6:** Correlation of high and low RIPASA scores with histopathology RIPASA: Raja Isteri Pengiran Anak Saleha Appendicitis, TP: true positive, FP: false positive, FN: false negative, TN: true negative

RIPASA score	Histopathology positive	Histopathology negative	Total
High score	32 (TP)	1 (FP)	33
Low score	2 (FN)	15 (TN)	17
Total	34	16	50

Both the RIPASA and MA systems were compared using various statistical parameters as shown in Table 7. The sensitivity was 94.11% vs 70.58%, the specificity was 93.75% vs 68.75%, PPV was 96.96% vs 82.75%, NPV was 88.23% vs 52.38%, and NAR was 2% vs 10% using the RIPASA vs MA score, respectively.

**Table 7:** Comparison between RIPASA and MA scores MA: modified Alvarado, RIPASA: Raja Isteri Pengiran Anak Saleha Appendicitis, PPV: positive predictive value, NPV: negative predictive value, NAR: negative appendectomy rate, TP: true positive, FP: false positive, FN: false negative, TN: true negative

Statistical parameters	MA scoring system	RIPASA scoring system	p-value
Sensitivity [TP/(TP+FP)]	70.58%	94.12%	0.0001, significant
Specificity [TN/(TN+FN)]	68.75%	93.75%	0.0001, significant
PPV [TP/(TP+FN)]	82.75%	96.96%	0.001, significant
NPV [[TN/(TN+FP)]	52.38%	88.23%	0.0001, significant
NAR	10%	2%	0.01, significant

There was a statistically highly significant correlation of all parameters when using the RIPASA vs the MA score as shown in Table 7.

## Discussion

Acute appendicitis is a very common presenting emergency, with an annual incidence of 96.5 to 100 cases per 100000 adults [9]. It requires surgical intervention in the form of open or laparoscopic appendectomy for its definitive management. The diagnosis can often be misleading and give way to unnecessary surgical interventions called negative appendectomy. With changing times and with advancements in technology, various diagnostic modalities have been used to assist in the diagnosis and to reduce NAR. However, for a surgeon, clinical acumen is the one that is most relied on. In addition, the lack of facilities and expertise in resource-poor countries makes clinical diagnosis of paramount importance. Thus, various clinical scoring systems were developed to rule out unnecessary surgical interventions. The MA score was used widely in the Western population with proven efficacy [6]. The RIPASA score was developed for better accuracy for Asian and Middle Eastern populations [7]. This study was conducted to compare the predictivity and preventability of NAR by using the RIPASA and MA scores.

In this study, a total of 50 operated patients of acute appendicitis as per surgeon discretion were included, and the scores were recorded according to both the clinical scoring systems. There were 68% (34/50) of patients operated on and who were noted to have an accurate diagnosis, whereas 32% (16/50) of patients did not have appendicitis.

The most common age group affected was the 15-30 years age group with 46% (23/50) of patients, followed by 26% (13/50) in the 31 to 45 years, 14% (7/50) in the 46 to 60 years, and 8% (4/50) patients in the less than 15 years. The least common age group affected was 61 to 75 years with 6% (3/50) of patients. Thus, the incidence is more common in young adults compared to extremes of age. This resembles the study by Oguntola et al. [10].

Males were twice (66%, 33/50) as commonly affected compared with females (34%, 17/50) similar to the study by Stein et al. [11]. There were five false positive cases in this study using the MA score. Thus, the NAR was found to be 10%. Numerous studies reported NAR between 6% and 44% using the MA score as shown in Table 8.

**Table 8:** Comparison of various studies for NAR using the MA score NAR: negative appendectomy rate

Sr no.	Study	NAR (%)
1	Sridhar et al. [12]	6
2	Patel et al. [13]	12.5
4	Prabhu et al. [14]	21
5	Kothari et al. [15]	32.5
3	Mitra et al. [16]	44
6	Current study	10

Since there was a vast difference in NAR as seen in various studies, it is observed that the MA score was not precise in detecting acute appendicitis.

There was one false positive case when the RIPASA score was used. Thus, the NAR was found to be 2%. Numerous studies conducted using the RIPASA score showed NAR between 0.7% and 17.39% as shown in Table 9.

**Table 9:** Comparison of various studies for NAR using the RIPASA score NAR: negative appendectomy rate

Sr no.	Study	NAR (%)
1	Karan et al. [17]	0.7
2	Chong et al. [7]	6.9
3	Abdelrhman et al. [18]	11
4	Rathod et al. [19]	17.39
5	Current study	2

Thus, our study showed a significant reduction (p < 0.01) in the NAR from 10% to an acceptable rate of 2%, similar to the various studies mentioned above. Thus, it is evident that the NAR is low with the RIPASA scoring system (0.7-17.39%) vs with the MA scoring system (6-44%).

Limitations

As patients with unlikely scores were not operated on and, hence, could not be included in the study, we need to further find methods to identify true cases of acute appendicitis in these patients using different modalities like CECT abdomen to identify patients of acute appendicitis with unlikely scores and clinical suspicion of acute appendicitis.

## Conclusions

Acute appendicitis is the most common condition needing surgical management with high NAR despite significant improvements in clinical and diagnostic modalities. Numerous clinical scoring methods have been devised to reduce NAR. The RIPASA score is a significantly better, specific, and cost-effective scoring system for diagnosing acute appendicitis and reducing NAR compared to the MA scoring method.
